# Flexible and Dynamic Scheduling of Mixed-Criticality Systems

**DOI:** 10.3390/s22197528

**Published:** 2022-10-04

**Authors:** Xiaowen Jiang, Tianyi Sha, Dehong Liu, Junjian Chen, Chen Chen, Kai Huang

**Affiliations:** 1 Institute of VLSI Design, Zhejiang University, Hangzhou 310027, China; 2 Digital Grid Research Institute, China Southern Power Grid, Guangzhou 510670, China

**Keywords:** mixed-criticality system, task scheduling, resource allocation, service level

## Abstract

A mixed-criticality system refers to an integrated embedded system in which tasks with different criticality levels run on a shared computing platform. In the design and development of mixed-criticality systems, how to schedule tasks to ensure that high-criticality tasks are executed in time and low-criticality tasks are served as much as possible is a major problem to be studied. Existing studies tend to consider pessimistic processing strategies to ensure the schedulability of functional tasks with high-criticality requirements. However, excessive pessimistic processing can lead to waste of system resources, thereby reducing the performance of functional tasks with low-criticality requirements. In this paper, we propose an adaptive-service-level adjustment strategy for low-criticality tasks, which solves the problem of waste of resources caused by invalid compensation in the low-criticality task compensation method of flexible mixed-criticality systems. In view of the problem that the existing methods mostly use static budget allocation and static independent mode switching without considering the actual operation of the task, this paper also proposes a flexible and dynamic mixed-criticality system scheduling scheme and designs a system execution framework, scheduling algorithm, and dynamic allocation strategy of maximum execution budget, in order to reduce unnecessary redundant resource expenditures and system switching costs and to improve the performance of low-criticality tasks. Experiments show that the proposed methods are effective compared to the state-of-the-art.

## 1. Introduction

The continuous breakthroughs in integrated circuit technology have greatly improved the scale and performance of embedded systems. In recent years, mixed-criticality systems have been increasingly used in real-time embedded fields such as automobiles, power electronics, and aerospace [1,2,3]. For example, in the field of smart grids, there is a mixed-criticality system of power inspection drones composed of computing tasks with different safety requirements, such as motor control and image acquisition. Whether the motor control function calculation task can be safely and reliably scheduled will directly affect the flight of the inspection drone. If such high safety-demand computing tasks cannot be executed in time, the impact on the inspection drone system will be serious or even fatal. However, the failure to perform computing tasks with low safety requirements, such as taking photos and collecting photos in a timely manner, will affect the quality of the fault inspection photos obtained by the grid personnel and will have less impact on the inspection UAV system.

In a mixed-criticality system, computing tasks of functional subsystems with different criticality are represented by different criticality levels, and tasks are divided into high-criticality tasks and low-criticality tasks according to the level of safety requirements [1]. Such a system has two modes: high-criticality mode (HC) and low-criticality mode (LC). When a high-criticality task overruns, the system will switch to HC mode and the task will switch to the high-criticality level (HI). To ensure the schedulability of high-criticality tasks, existing works [4,5,6,7,8,9,10,11] mainly include the following three aspects:

(1) Ensure that high-criticality tasks are executed in a timely manner: that is, directly abandon the execution of all low-criticality tasks, which will reduce the service quality of low-criticality tasks, thereby affecting the user’s overall experience of functional requirements;
(2) Static task mode switching: that is, when a high-criticality task overruns, all other high-criticality tasks will switch to HC mode, which generates unnecessary resource budgets and wastes system resources;
(3) Static budget allocation method: that is, the execution resource overhead of high-criticality tasks in LC mode is determined before the task is scheduled and the execution resources are allocated directly according to the execution time of high-criticality tasks in LC mode.

Many researchers have studied the above problems and proposed solutions. In the work [12,13,14,15,16,17,18,19,20], once the high-criticality task fails to execute in time in LC mode, the low-criticality tasks will not be discarded directly, but their service levels are degraded to different degrees by adjusting the frequency allocation or execution time budget of the low-criticality tasks. This can balance the greater execution overhead required by the high-criticality task switching to HC mode. Aiming at the shortcomings of the second aspect above, the studies [21,22,23,24,25,26,27,28,29] proposed different mode switching triggering methods, which improved the problem of a large unnecessary resource pre-sale caused by the static mode switching. It is worth mentioning that the authors in [30] proposed a flexible mixed-criticality (FMC) system optimization scheme. However, this approach is very conservative. In fact, it is extremely unlikely that all high-criticality tasks will overrun at the same time. When not all high-criticality tasks have overruns, the system can give more idle resources to compensate for overruns of high-criticality tasks. For such a demand compensation calculation method, the FMC scheme does not consider that when there are redundant execution resources in the system, the idle resources are recovered and compensated for the low-criticality tasks that are degraded due to the overrun of the high-criticality tasks. Therefore, the scheme has the problem of invalid compensation for a large number of low-criticality tasks, which greatly reduces the execution performance of low-criticality tasks. In addition, the method adopts a static budget allocation method and does not consider the actual operation of high-criticality tasks. When the actual execution time of high-criticality tasks is much less than the estimated execution time, there will be significant redundant resource expenditures, resulting in the waste of system resources and performance degradation of low-criticality tasks.

To the best of our knowledge, none of the existing research has solved the problem of ineffective compensation of low-criticality tasks and has not considered the solution of reasonably allocating system resource overhead according to the actual operating conditions of high-criticality tasks in LC mode. This paper proposes a novel dynamic mixed-criticality system task scheduling scheme, which reduces unnecessary system pessimism while ensuring the schedulability of high-criticality tasks and realizing the optimization of task schedulability and system switching cost in mixed-criticality systems. Specifically, this paper proposes a more practical adaptive service level adjustment strategy for low-criticality tasks. The system can dynamically downgrade the service level of low-criticality tasks by adjusting the execution frequency of low-criticality tasks according to the overspending situation of high-criticality tasks, which solves the problem of invalid compensation for low-criticality tasks and has better performance in supporting the execution of low-criticality tasks. This paper also proposes a task scheduling scheme for dynamic mixed-criticality systems. The system can dynamically allocate resources according to the actual execution of high-criticality tasks in LC mode, reducing unnecessary waste of resources caused by pessimistic processing. The simulation results show that the proposed scheme has obvious optimization effects compared with the existing state-of-the-art schemes in terms of task schedulability and system switching cost.

The rest of this paper is organized as follows. Section 2 introduces related work and Section 3 presents typical models of mixed-critical systems. Section 4 presents an example of research motivation. Section 5 introduces the method proposed in the work and Section 6 is the simulation experiment and result analysis. Section 7 concludes the paper.

## 2. Related Work

In a mixed-criticality system, tasks are divided into high-criticality tasks and low-criticality tasks according to safety requirements. Tasks have different execution time budget estimates at their respective criticality levels. By default, all tasks are in LC mode. The high-criticality tasks are scheduled with smaller execution time estimates and low-criticality tasks are scheduled with fixed execution time estimates. Once a high-criticality task overruns and switches to HC mode, it will be scheduled with a larger execution time estimate. Most of the existing algorithms are extensions based on the nearest deadline first algorithm proposed in traditional real-time systems. In order to ensure the schedulability of high-criticality tasks, the strategies adopted in research [4,5,6,7,8,9,10,11] are: if any high-criticality task fails to complete the scheduling in time within its execution time budget estimate, then all high-criticality tasks are switched to HC mode. At the same time, more execution resources are given to high-criticality tasks to ensure the schedulability of high-criticality tasks. All low-criticality tasks will be suspended immediately to balance the execution overhead of switching high-criticality tasks to HC mode. For example, Baruah combined the earliest-deadline first (EDF) algorithm and proposed a mixed-criticality scheduling algorithm called the earliest-deadline first virtual deadline (EDF-VD) [5]. Su et al., proposed a strategy to set variable execution periods (i.e., service intervals) for low-criticality tasks [14]. In this strategy, when the system switches to HC mode, the low-criticality task reduces its allocation frequency so that the quality of service of the low-criticality task is reasonably degraded instead of being discarded directly, and its minimum service requirement is guaranteed by its maximum cycle. Low-criticality tasks can also reclaim the idle time generated by the redundant execution time budget of high-criticality tasks, and release them in advance to shorten the service interval, thereby improving their service level. Liu et al. proposed a scheme from the perspective of reducing the execution time budget of low-criticality tasks [15]. However, the above research still adopts the mode switching that depends on the triggering method and the static budget allocation method.

Ren and Phan proposed a partition scheduling method for grouped Pfair-like scheduling [18]. In a task group, a high-criticality task encapsulates several low-criticality tasks. The tasks of each task group are decomposed into quantum-length subtasks, and the subtasks of different groups are scheduled on the basis of earliest-pseudo-deadline-first (EPDF). Although using Pfair scheduling can delay mode switching and support low-criticality tasks with better execution performance, it also brings many practical problems. For example, decomposing into quantum-length subtasks for scheduling results in very high scheduling overhead due to frequent preemption, limited scalability due to NP-hard complexity, etc. Gu et al. proposed a component-based strategy in which component boundaries provide the isolation needed to support low-criticality task execution [22]. Slight overruns can be handled by internal mode switching by shutting down all low-criticality tasks within the component. A wider overrun would result in a system-wide external mode switch and interruption of all less critical tasks. Although this strategy has a certain ability to delay mode switching and reduce unnecessary waste of resources, it still adopts the pessimistic process that once the high-criticality task overruns, all low-criticality tasks immediately give up execution. Furthermore, the strategy tests the schedulability of the system using a demand constraint function-based approach. The complexity of schedulability tests scales exponentially with the size of the input, leading to computationally expensive issues.

The latest research [30] presented an FMC system optimization scheme in which the mode switching of the high-criticality tasks is triggered independently and the tasks do not affect each other. Any high-criticality task overrun will only trigger itself to switch into HC mode and other high-criticality tasks will remain in the original mode. At the same time, the system will downgrade the service level of low-criticality tasks according to the overrun of high-criticality tasks, and will not immediately abandon the execution of all low-criticality tasks. However, the demand compensation calculation used for the service level degradation of the low-criticality tasks is carried out by considering the way of distributing the idle resources of the system in equal proportions in the case that all the high-critical tasks will overrun.

## 3. System Model

The system studied in this paper has high-criticality level (HI) and low-criticality level (LO), and the criticality level of the system in the initial state is LO. In this system, a total of n mixed-critical period real-time tasks are scheduled to run, and the set composed of these tasks is denoted as $\gamma =\{\tau_{1},\tau_{2},\ldots ,\tau_{n}\}$ for each mixed-critical period task $\tau_{i}$, denoted as $\tau_{i}=\left\{T_{i},L_{i},C_{il},C_{ih}\right\}$, where $T_{i}$ represents the period of the task $\tau_{i}$ and also serves as the relative deadline of the task in this model, i.e., $T_{i}=D_{i}$. The vector $L_{i}=\left\{LO,HI\right\}$ represents the critical level of task $\tau_{i}$; $L_{i}=HI$ means that the task is a high-criticality task and $L_{i}=LO$ means that the task is a low-criticality task. $C_{il}$ and $C_{ih}$ represent the worst case execution time (i.e., WCET) budget estimates for task $\tau_{i}$ in LC mode and HC mode, respectively. High-criticality tasks have different WCET budget estimates in LC mode and HC mode, satisfying $C_{il}<C_{ih}$, and low-criticality tasks have only one WCET budget estimate in LC mode and HC mode, satisfying $C_{il}=C_{ih}$.

In order to better characterize the execution budget of low-criticality tasks in HC mode, we introduce the following definitions:

**Definition** **1** (**k-level HC mode**). *At a given moment, if k high-criticality tasks switch to HC mode, the system is in k-level HC mode. When all high-criticality tasks are in LC mode, the system can be considered to be in 0-level HC mode.*

**Definition** **2** (**service level**). *The service level is used to represent the service quality of the low-criticality tasks after the mode switching of the high-criticality tasks, which is denoted here as $Z_{i}^{k}$. Each time the system switches modes, the current service level will be updated to determine the new service quality of low-criticality tasks. Assuming the initial state $Z_{i}^{0}=1$ and $Z_{i}^{k}\geq Z_{i}^{k-1}$, if the system has experienced the $k_{th}$ mode transition at this time, the new service quality of the low-criticality task $\tau_{i}$ is $Z_{i}^{k}\cdot T_{i}$, that is, the execution frequency of the low-criticality task is adjusted from $Z_{i}^{k-1}\cdot T_{i}$ to $Z_{i}^{k}\cdot T_{i}$.*

**Definition** **3** (**k-level maximum execution resource budget**). *The k-level maximum execution resource budget refers to the maximum resource expenditure for which all high-criticality tasks in LC mode can still run in LC mode when the system is in k-level HC mode, which is denoted here as $u_{threshold}^{k}$. Assuming that the 0-level maximum execution resource budget is the sum of the estimated execution time budget of all high-criticality tasks in LC mode for the system CPU utilization (denoted as $u_{ihl}$), for the entire task set, the 0-level maximum execution resource budget is:*

(1) $$u_{threshold}^{0}=\sum_{\tau_{i}\in \gamma_{HI}}u_{ihl}$$

The *k*-level maximum execution resource budget can be obtained by subtracting the cost of high-criticality tasks in LC mode before switching to HC mode from the 0-level maximum execution resource budget. When the system is in the k-level HC mode, once the remaining high-criticality task execution resource overhead in LC mode exceeds the *k*-level maximum execution resource budget, a new high-criticality task will be triggered to perform mode switching and the HC mode of the system will be increased by one level. The all *k*-level maximum execution resource budget is:

(2) $$u_{threshold}^{k}=\sum_{\tau_{i}\in \gamma_{HI}}u_{ihl}-\sum_{\tau_{i}\in \gamma_{HI}^{HI}}u_{is}$$

Here, $u_{is}$ refers to the execution resources occupied by the high-criticality task $\tau_{i}$ in LC mode before switching to HC mode. $\gamma_{HI}^{HI}$ refers to the high-criticality task set in HC mode. In addition, the calculation formulas of other related utilization rates are as follows.

The resource utilization of tasks in different modes is:

(3) $$u_{iab}=\frac{C_{ib}}{T_{i}}$$

*a* indicates whether the type of task is high-criticality (h) or low-criticality (l). *b* indicates the type of system mode, which is either high-criticality mode (h) or low-criticality mode (l).

For example, the resource utilization of high-criticality tasks in LC mode is:

(4) $$u_{ihl}=\frac{C_{il}}{T_{i}}$$

The resource utilization of low-criticality tasks after the $k^{th}$ independent mode transition is:

(5) $$u_{ill}^{k}=\frac{C_{il}}{T_{i}\times z_{i}^{k}}$$

For the entire task set, the resource utilization of tasks is:

(6) $$u_{ab}=\sum_{\tau_{i}\in \gamma_{a}}u_{iab}$$

For example, the resource utilization of high-criticality tasks in HC mode is:

(7) $$u_{hh}=\sum_{\tau_{i}\in \gamma_{HI}}u_{ihh}$$

The resource utilization of low-criticality tasks after the *k*-th independent mode transition is:

(8) $$u_{ll}^{k}=\sum_{\tau_{i}\in \gamma_{LO}}u_{ill}^{k}$$

The resource utilization at minimum quality of service for low-criticality tasks is $u_{lO}^{man}=\sum_{\tau_{i}\in \gamma_{LO}}z_{i}^{man}u_{ill}$, where $z_{i}^{man}$ is the minimum service level specified by the user.

## 4. Motivation Case

When tasks are executed on a mixed-criticality system, the key is how to ensure the timely execution of high-safety-critical tasks while enabling low-safety-critical tasks to be served as much as possible. Table 1 lists the basic parameters of each task. In this example, there are a total of six mixed-critical tasks, of which $\tau_{2}∼\tau_{4}$ are high-criticality tasks and the rest are low-criticality tasks. $D_{i}^{{}^{\prime }}$ represents the virtual deadline obtained by multiplying the virtual time factor $D_{i}$, and $D_{i}^{{}^{\prime }}$ is only applicable when the high-criticality task is in LC mode. A low-criticality task has only one WCET estimate in both LC mode and HC modes, and satisfies $C_{il}=C_{ih}$.

**Figure 1:**
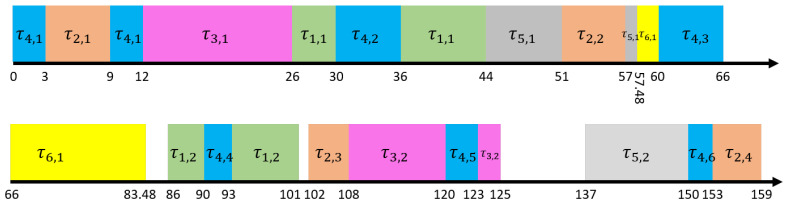
Scheduling result of task set under FMC scheme.

Figure 1 shows the scheduling result of the task set under the FMC scheme. According to the scheme, the first job of task $\tau_{4}$ (denoted as $\tau_{4,1}$) is first selected for scheduling at time 0. When the system runs to time 3, the high-criticality job $\tau_{4,1}$ fails to complete its execution within its worst execution time estimate (3 time units); i.e., the high-criticality job overruns and switches to HC mode. The resource requirements of the overrun part of the high-criticality tasks are compensated according to the principle of the minimum task utilization priority in the FMC scheme. Since the low-criticality task $\tau_{5}$ has the smallest utilization rate, the low-criticality task $\tau_{5}$ will be selected first to compensate; that is, the task execution time budget needs to be adjusted from the original 17 to 7.54. As long as the low-criticality job $\tau_{5,1}$ cannot be completed within 7.54 time units, the job fails, and the next job can only be executed after waiting for the time budget of the next cycle.

However, as can be seen from Figure 1, the deadline for the low-criticality job $\tau_{5,1}$ is 137 and at time 137, there are still a number of abundant idle resource blocks in the system. If these idle resources can be utilized, the low-criticality job $\tau_{5,1}$ can actually be scheduled according to the original execution time budget without reducing the execution time budget. Therefore, the FMC scheme does not consider the recovery of the system’s idle resources and inversely compensates for the low-criticality tasks whose execution time is reduced due to the overrun of the high-criticality tasks when the system has redundant execution resources that can be recovered.

**Figure 2:**
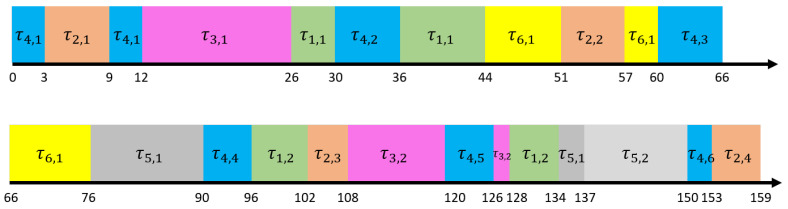
Scheduling results of task sets under the improved FMC scheme.

Figure 2 shows the final scheduling result of the task set under the improved FMC scheme. Different from Figure 1, the resource requirement of the overrun portion of switching the high-criticality task $\tau_{4}$ to HC mode is realized by reducing the execution frequency of the low-criticality task $\tau_{5}$. The new execution period of low-criticality task $\tau_{5}$ is extended from the original 137 time units to 311 time units and its execution time budget, which is still 17 time units. is not reduced. It can be seen from the final scheduling result in Figure 2 that the idle system resources can be better utilized under this scheme, and the low-criticality tasks $\tau_{5,1}$ has been executed within 137 time units of its original specified time and the execution budget has not been reduced.

## 5. FMCI and FDMC Schemes

To address the two problems in the traditional mixed-critical system model, namely, once any high-criticality task overruns, all low-criticality tasks are suspended, and all other high-criticality tasks are assumed to exhibit high-criticality behavior at the same time, ref. [30] has proposed a more realistic mixed-criticality model, called the flexible mixed-criticality (FMC) model. In FMC, it is assumed that only the overrun task itself exhibits high-critical behavior, while the other high-criticality tasks maintain the same mode as before and the guaranteed service level of the low-critical task degrades relative to the high-critical task.

However, the problem of invalid compensation of low-criticality tasks occurs in the existing scheme because the demand compensation calculation is only for the most pessimistic scheduling situation; in non-pessimistic cases, the use of system idle resource recovery to reversely compensate low-criticality tasks is not considered. In addition, the existing scheme adopts the methods of static budget allocation and static mode switching. The execution resources are specified before tasks are scheduled and the actual operation of the tasks is not considered. However, in actual task scheduling, if the actual execution time of a high-criticality task is much smaller than its worst execution time budget estimate, it will cause significant waste of resources. In response to the above problems, this paper proposes an adaptive service level adjustment method for low-criticality tasks, namely FMC improved (denoted as FMCI), which can enable low-criticality tasks to have better execution performance. This paper also proposes a more practical feasible dynamic mixed-criticality system (denoted as FDMC), which can dynamically allocate resources according to the actual operation of high-criticality tasks in LC mode, reducing unnecessary resources caused by pessimistic processing.

### 5.1. FMCI

In the FMC scheme [30], the demand compensation calculation is shown in Formula (9). $u_{ll}^{k}$ is the service level of a low-criticality tasks in the k-level HC mode. $\Delta u_{ll}$ is the minimum compensation overhead adjustment value that a low-criticality task needs to make to balance the resource requirements of high-criticality task overrun when the system switches from the k-1 level HC mode to k-level HC mode.

(9) $$\Delta u_{ll}=u_{ll}^{k}-u_{ll}^{k-1}\leq min\left(0,\frac{u_{ihl}/u_{hl}\times \left(1-u_{ll}\right)-u_{ihh}}{1-x}\right)$$

From Formula (9), it can be seen that the dynamic adjustment of service quality of low-criticality tasks is analyzed based on the technology of utilization, and the following two points can be observed.

(1) When a new high-criticality task mode switch occurs, the low-criticality task service dynamic adjustment strategy will adjust the utilization rate of its own tasks according to the overrun of the high-criticality task to compensate.

(2) The adjustment of the utilization rate of the low-criticality task is only determined by the high-criticality task where mode switching occurs. This means that the mode switching of high-criticality tasks has an independent effect on adjusting the utilization of low-criticality tasks, and the sequence of mode-switching of high-criticality tasks has no effect on the adjustment of service levels of low-criticality tasks.

The change in utilization is directly affected by two factors: execution time and execution frequency (the reciprocal of the execution cycle). Utilization is positively correlated with execution time and negatively correlated with execution frequency. Therefore, in addition to reducing the execution time budget of low-criticality tasks, service quality degradation of low-criticality tasks can also be achieved by extending the execution frequency of low-criticality tasks.

Aiming at the shortcomings of the low-criticality task service adjustment compensation method in the FMC scheme, this paper presents a more practical low-criticality task adaptive service level adjustment scheme. The system downgrades the service level of low-criticality tasks according to the overrun of high-criticality tasks, and the resource requirements of the over-expenditure part are met by reducing the execution frequency of low-criticality tasks, not by reducing the execution time of low-criticality tasks. The improved calculation method of demand compensation is shown in Formula (10), where $\Delta u_{ll}$ is the minimum compensation overhead adjustment required by the low-criticality task to balance the resource requirements of the overrun portion of the high-criticality task, when the system switches from the k-1 to the k level HC mode. $T_{i}^{k}$ is the execution period of the low-criticality task $\tau_{i}$ in the k-level HC mode ($T_{i}^{0}=T_{i}$). $\Delta T_{ll}$ is the execution period that needs to be elongated, i.e., the execution frequency that needs to be reduced. The low-criticality task service level needs to adjust its execution frequency to achieve compensation for the overrun resource requirements of the high-criticality task, which can be calculated by (10). In the adjustment scheme proposed in this paper, the execution time of low-criticality tasks will not be reduced, so the situation of ineffective compensation of low-criticality tasks can be avoided and the service level of low-criticality tasks can be better dynamically reduced.

(10) $$\Delta u_{ll}=u_{ll}^{k}-u_{ll}^{k-1}=\frac{c_{il}}{T_{i}^{k}}-\frac{c_{il}}{T_{i}^{k-1}}\Rightarrow \Delta T_{ll}=T_{i}^{k}-T_{i}^{k-1}=\frac{-{\left(T_{i}^{k-1}\right)}^{2}\times \Delta u_{ll}}{T_{i}^{k-1}\times \Delta u_{ll}+c_{il}}$$

### 5.2. FDMC

In FDMC, high-criticality tasks will dynamically allocate system resources according to their actual execution time requirements in LC mode and will no longer be statically allocated according to the estimated execution time budget that has been allocated before scheduling. The conditions for switching high-criticality tasks to HC mode in LC mode are no longer based on their execution time budget estimates, but are dynamically evaluated based on the actual execution of all high-criticality tasks in LC mode. This section introduces the execution framework, scheduling algorithm, and maximum execution budget allocation strategy involved in FDMC.

#### 5.2.1. Execution Framework

**Figure 3:**
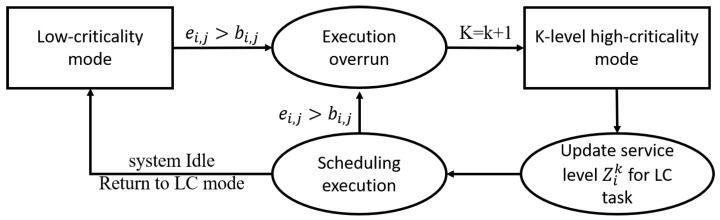
FDMC execution framework.

Initially, all tasks are in LC mode. In the scheduling execution process, once any high-criticality job overruns, it will be triggered to switch to HC mode, and the level of the system HC mode will also be increased by one level. If the conditions for returning to LC mode are met during the scheduling process, the task will return to LC mode again, and so on until the scheduling ends. The execution framework is shown in Figure 3.

LC mode: In the initial state, all tasks are in LC mode. As long as the maximum execution budget $u_{threshold}^{0}$ is not exceeded, the system can always maintain task scheduling in LC mode.

Scheduling and execution: During the scheduling process, when a new job is released, it will be inserted into the task queue to be scheduled and the system will select the job with the highest priority in the task queue to be scheduled. When job $\tau_{i,j}$ is a high-criticality job and cannot be completed within $b_{i,j}$, it immediately switches from LC mode to HC mode; other high-criticality tasks remain unchanged and the system HC mode level k is raised by one level.

Update low-criticality tasks service level: During the scheduling and execution process, the system will update the current service level $Z_{i}^{k}$ of the low-criticality task every time the system switches modes, and the low-criticality task execution frequency is adjusted from $Z_{i}^{k-1}\cdot T_{i}$ to $Z_{i}^{k}\cdot T_{i}$. Changes in $Z_{i}^{k}$ are dynamically adjusted by the specific overruns of high-criticality tasks. Under the new HC mode level, the low-criticality job execution time budget remains unchanged and is scheduled according to the new deadline.

Return to LC mode: When the system detects an idle state, that is, when there are no more tasks to be scheduled, the system returns to LC mode, and the execution frequency of low-criticality tasks returns to $T_{i}$.

#### 5.2.2. Maximum Execution Budget Allocation Strategy

Aiming at the problem that the static budget allocation method of high-criticality tasks in LC mode is not flexible and practical in mixed-criticality system scheduling, this section proposes a maximum execution budget allocation strategy. In the FDMC scheme, the execution time overhead of high-criticality tasks in LC mode is dynamically allocated according to the actual execution time of their running. The overall idea of the maximum execution budget allocation strategy is to ensure that high-criticality tasks can dynamically allocate execution overhead according to the actual time required for their scheduled execution in LC mode and, at the same time. evaluate the upper limit of the execution budget with which high-criticality tasks can keep running in LC mode to ensure the schedulability of the high-criticality tasks. That is, if the actual time required for its scheduled execution is greater than the maximum execution budget that can be allocated by the calculation of the maximum execution budget allocation strategy, then the high-criticality task needs to be switched to HC mode immediately and the other high-criticality tasks remain in the original mode. In this way, the actual situation of task scheduling is more realistically considered, and unnecessary waste of resources can be reduced; the frequency of system mode switching can also be delayed, finally ensuring the schedulability of high-criticality tasks and improving the execution performance of low-criticality tasks.

**Figure 4:**
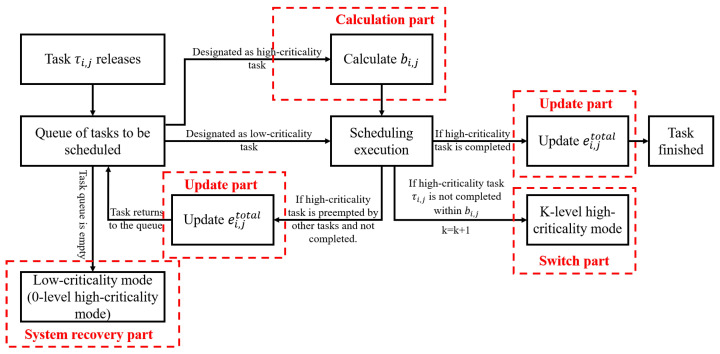
The maximum execution budget allocation strategy.

The following describes how the maximum execution budget allocation strategy in the FDMC scheme supports the dynamic allocation of execution overhead for high-criticality tasks in LC mode. As shown in Figure 4, it can be divided into four parts.

(1) Calculation: when the currently scheduled job is selected from the task queue to be scheduled as a high-criticality job, calculate the maximum execution time budget $b_{i,j}$ that can be allocated by the scheduling job $\tau_{i,j}$ in LC mode according to Formula (11); then, use it as an indicator of whether to trigger the switch to HC mode. When the task assigned by the current scheduling is selected from the task queue to be scheduled as a low-criticality task, this calculation link is not required.

(11) $$b_{i,j}=T_{i}\times \left(u_{threshold}^{k}-\sum_{\tau_{n}\in \gamma_{HI}^{LO}}\frac{e_{n,j}^{total}}{T_{n}}\right)$$

(2) Switching: When the high-criticality job $\tau_{i,j}$ fails to complete within its maximum execution time budget $b_{i,j}$, a mode switch will be triggered; then, it will switch from LC mode to HC mode and the value of k-level HC mode increases by one. Excessive execution of this task will not trigger other high-criticality tasks to enter HC mode. In the latest HC mode level, the system re-evaluates the execution frequency of low-criticality tasks according to the overrun of high-criticality tasks to balance the resource requirements caused by over-expenditure of high-criticality tasks. At the same time, the execution time budget of low-criticality tasks remains unchanged and is still scheduled according to the new deadline. Finally, at this latest HC mode level, the maximum execution resources are also recalculated to evaluate if the remaining high-criticality tasks that have not switched to HC mode can remain running in LC mode.

The mode switching conditions are as follows. Assuming that the system is in a busy state at time $t^{\circ }$, and job $\tau_{i,j}$ is being scheduled, the mode switching of the system is triggered at this time. $e_{i,j}^{t}$ represents the total execution time of the $|\frac{t}{T_{i}}|+1$ -th job of task $\tau_{i}$ before time t. If the scheduling of job $\tau_{n,j}$ starts from time t and an overrun occurs at time $t^{\circ }$, then it must satisfy:

(12) $$\sum_{\tau_{i}\in \gamma_{HI}^{LO}}\frac{e_{i,j}^{t}}{T_{i}}⩽u_{threshold}^{k}$$

(13) $$\sum_{\tau_{i}\in \gamma_{HI}^{LO}}\frac{e_{i,j}^{t^{\circ }}}{T_{i}}=u_{threshold}^{k}$$

(3) Update: The maximum execution time budget $b_{i,j}$ that can be allocated to a high-criticality job is related to the actual execution time $e_{n,j}^{total}$ of other high-criticality jobs $\tau_{n,j}$. Therefore, when each high-criticality job $\tau_{i,j}$ is completed or preempted by a higher priority job and needs to be returned to the queue to be scheduled, the update of $e_{i,j}^{total}$ is triggered and the duration $e_{i,j}$ of the current scheduling operation is accumulated into $e_{i,j}^{total}$.

(4) Recovery: When the system detects an idle state, that is, there are no more tasks to be scheduled, the system recovery process is triggered. All tasks return to LC mode, A is cleared, and the execution frequency of the low-criticality task returns to the original execution frequency.

#### 5.2.3. Scheduling Algorithm

This section introduces the algorithm based on EDF-VD scheduling suitable for the FDMC scheme, which is called the FDMC-EDF-VD algorithm. Before the scheduling starts, the virtual deadline factor *x* is obtained by calculating $\frac{u_{hl}}{1-u_{ll}}$. Formula (14) is the test condition to ensure the schedulability of the task under FDMC-EDF-VD. $u_{threshold}^{0}$ is the maximum resource cost under which all high-criticality tasks can keep running in LC mode, and us used to determine whether the system needs to switch to HC mode in the online task scheduling execution stage.

(14) $$\left(1-x\right)\left(u_{ll}-u_{ll}^{man}\right)+\sum_{\tau_{i}\in \gamma_{HI}}\phi \left(\tau_{i}\right)\geq 0$$

(15) $$\phi \left(\tau_{i}\right)=\frac{u_{ihl}}{u_{threshold}^{0}}\left(1-u_{ll}\right)-u_{ihh}\left(\tau_{i}\in \gamma_{HI}\right)$$

In the initial state of scheduling, all high-criticality tasks will be scheduled according to the virtual deadline $x\times T_{i}$. When any high-criticality task overruns, it will immediately switch to HC mode. The deadline used for scheduling the overrun high-criticality task is restored from the original virtual deadline $x\times T_{i}$ to the actual deadline $T_{i}$, and other high-criticality tasks are not affected and still keep the original deadline for scheduling. At the same time, the execution frequency of low-criticality tasks under the new service level is updated according to Formula (16), so as to achieve a utilization balance between low-criticality tasks and high-criticality tasks. Then, calculate the maximum execution resource budget in the latest HC mode according to Formula (2). During the scheduling process, when the system detects an idle state, that is, when there are no more tasks to be scheduled, the system returns to LC mode and the execution frequency of the low-criticality tasks returns to $T_{i}$ until the scheduling ends.

(16) $$\Delta u_{ll}=u_{ll}^{k}-u_{ll}^{k-1}\leq min\left(0,\frac{u_{ihl}/u_{threshold}^{0}\cdot \left(1-u_{ll}\right)-u_{ihh}}{1-x}\right)$$

The algorithm implementation framework is shown in Algorithm 1.
**Algorithm 1:** FDMC-EDF-VD
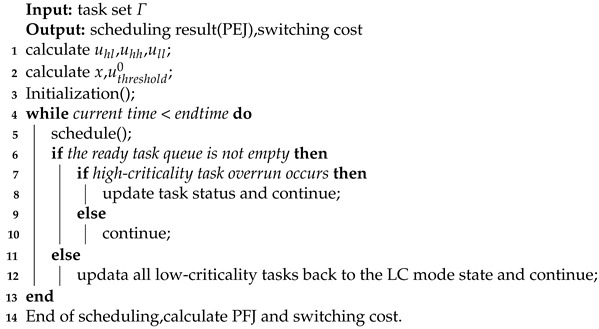


Lines 1–3 are the off-line calculation process before scheduling starts. First, according to Formulas (6) and (1), the utilization rates $u_{hh}$, $u_{hl}$, $u_{ll}$, and $u_{threshold}^{0}$ are calculated respectively, and the virtual deadline factor of the high-criticality task in LC mode is obtained. The initial state of all tasks is LC mode (Line 3). The task set enters the online scheduling stage when it satisfies the schedulability condition under FDMC-EDF-VD (i.e., Formula (11)). Lines 4–12 are the process of online scheduling. In LC mode, high-criticality tasks are scheduled according to their virtual deadlines. The system selects the task closest to the deadline from the queue of tasks to be scheduled for scheduling (Line 5). Once there is a high-criticality job $\tau_{i,j}$ that is not completed within the time $b_{i,j}$ (calculated dynamically based on the actual execution of all high-criticality tasks in LC mode) (Line 7), the task state immediately switches from LC mode to HC mode and receives a larger execution time budget, while other high-criticality tasks remain in their original mode. In this process, the overrun of the high-criticality task is calculated by Formula (15), the service quality of the low-criticality task is updated by Formula (16) to balance the resource demand, and the total maximum execution resource budget in the updated HC mode is also calculated according to Formula (2) for subsequent scheduling budget. When the assigned job is scheduled and there are no more jobs in the task queue to be scheduled (that is, the system detects idleness), the completion status of all tasks is calculated and updated, and all tasks return to LC mode; otherwise, continue to schedule the next job. Finally, when the scheduling is over, output the PFJ (indicating the completion rate of the successful execution of the low-criticality task within the deadline) and the result of the switching cost (Line 14).

## 6. Experiment and Evaluation

In this section, we evaluate the effectiveness of our proposed FMCI and FDMC through extensive simulations. All the simulations are implemented on the Visual Studio. The PC configuration information is Intel(R) Core(TM) I5-5200U CPU 2.20 ghz processor, memory: 6 GB.

Similar to the task settings in [5,8,22,30], the parameter settings of this experiment are configured first; then, the corresponding task set is generated. The parameters are as follows. The low-criticality utilization $u_{il}$ of each task is obtained by the UUnifast algorithm. The period $T_{i}$ of each task $\tau_{i}$ is an integer drawn randomly in the range [20,150] according to the average distribution. The ratio of resource utilization of high-criticality tasks in HC mode to LC mode is an integer drawn uniformly and randomly in [2,3]. The mixing critical level is set to L=2. The probability that a task is a high-criticality task is 0.5 and the lower bound $u_{ll}^{man}$ of the total utilization of a low-criticality task is 0.33 ([17] points out that most tasks will be completed within 33% of their estimated execution time, so we set the minimum service guarantee for low-critical tasks). In order to ensure the schedulability of the mixed-critical system, a limit $u_{B}$ of utilization is given here until the following two conditions are satisfied; if not, the task set is regenerated.

(1) $u_{B}-0.05\leq max\left(u_{ll}+u_{hl},u_{hh}\right)\leq u_{B}$;
(2) At least three high-criticality tasks are generated.

### 6.1. Simulation Verification of FMCI Scheme

**Figure 5:**
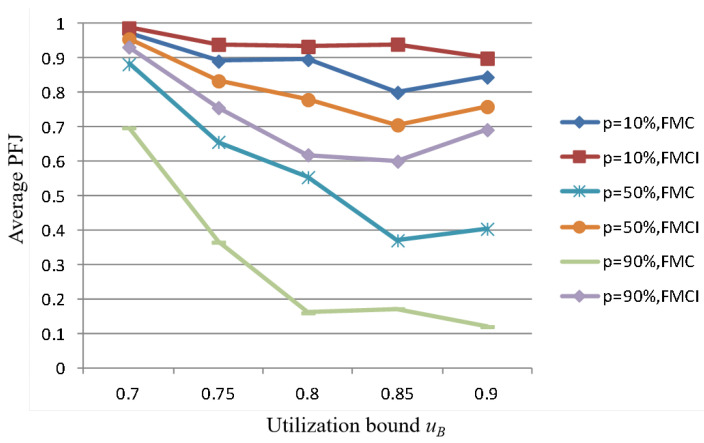
Comparison of scheduling results between FMCI and FMC.

We use the same test task set generated to perform task scheduling according to the FMCI scheme and the FMC scheme. Since the FMCI is an improved scheme to achieve computing resource compensation by reducing the execution frequency of low-criticality tasks, the PFJ here is represented by the ratio of the number of times that the low-criticality task actually successfully completes the scheduling to the number of times that the low-criticality task should complete the scheduling without changing its execution frequency. By comparing the results of PFJ, the performance of the two schemes in supporting the execution of low-criticality tasks while ensuring the schedulability of high-criticality tasks is evaluated. Since the generated mixed-critical task set has a certain degree of randomness, in order to obtain more reliable results, all data are the results of the average of 100 task sets scheduled. The parameter variables of the simulation experiment are the probability of overruns for high-criticality tasks ($u_{B}$), and the aggregate utilization rate of the generated tasks (*p*). In the case of different combinations of these two parameters (p∈0.1,0.3,0.5,0.7,0.9; $u_{B}\in 0.7,0.75,0.8,0.85,0.9$), the scheduling results corresponding to the FMCI scheme and the FMC scheme are shown in Figure 5. The horizontal axis represents the total utilization of the task set and the vertical axis represents the performance of low-criticality tasks (i.e., PFJ value).

It can be seen from Figure 5 that under the same conditions, the performance of low-criticality tasks under the FMCI scheme is always better than that of the FMC scheme. The performance of low-criticality tasks under the FMCI scheme is less volatile. This means that when the high-criticality task has a higher probability of entering HC mode or the scheduled task set is crowded, the performance of the low-criticality task is less affected.

**Figure 6:**
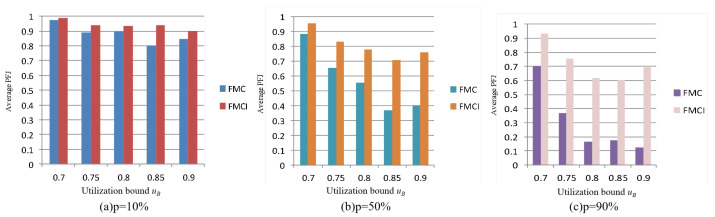
Comparison of scheduling results under different probabilities of overruns in high-criticality tasks.

The following is a further comparison of the differences between the FMCI scheme and the FMC scheme in the execution performance of low-criticality tasks under different probabilities (p=10%,p=50%,p=90%) of overruns in high-criticality tasks. As shown in Figure 6, with the increase of the probability of high-criticality tasks entering HC mode, the advantage of FMCI in the performance of low-criticality tasks will be more obvious.

### 6.2. Simulation Verification of FDMC Scheme

**Figure 7:**
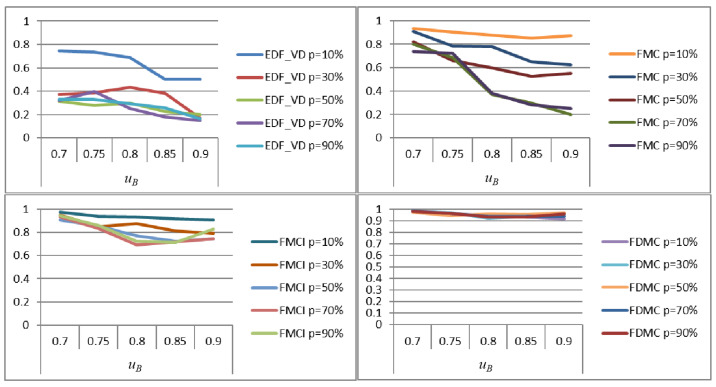
The PFJ evaluation of each scheme under different *p* values.

In this section, the simulation verification of the FDMC scheme is carried out. The PFJ results are also compared to evaluate the performance of the classic EDF-VD [5,7], FMC [30], FMCI, and FDMC for supporting low-criticality task execution while ensuring the schedulability of high-criticality tasks. Figure 7 shows the PFJ simulation experiment results of each scheme under different probabilities that the high-criticality task enters HC mode. The results are analyzed as follows.

Under the EDF-VD scheme, the execution performance of low-criticality tasks is the highest and the completion degree can reach more than 70%. The overall scheduling is greatly affected by the probability of high-criticality tasks switching to HC mode. When the probability of high-criticality tasks entering HC mode is above 30%, the scheduling completion of low-criticality tasks will drop significantly below 40%. When the high-criticality task has a high probability of entering HC mode or the generated scheduling task set is crowded, the execution performance of the low-criticality task will become very poor and the scheduling completion rate will be lower than 20%.

Under the FMC scheme, when the generated scheduling task set is not crowded, the execution completion degree of low-criticality tasks can reach 70%, while the highest can reach 93%. However, the overall scheme is greatly affected by the congestion degree of the generated scheduling task set and the probability of switching high-criticality tasks to HC mode. When the probability is greater than 70%, as the congestion degree of the scheduling task set increases, the execution completion degree of the low-criticality tasks will also drop significantly; the worst performance is lower than 20%.

Under the FMCI scheme, the execution performance of low-criticality tasks has a greater optimization effect in terms of stability and completion. The execution completion degree of low-criticality tasks is above 65% and the overall performance is less affected by the crowding degree of the generated task set and the probability of high-criticality tasks entering HC mode. When the probability of switching high-criticality tasks to HC mode is 10%, no matter how crowded the scheduling task set is, the completion degree of low-criticality tasks is stable above 90%.

Under the FDMC scheme, no matter the congestion degree of the generated task set and the probability of the high-criticality task entering HC mode change, the low-criticality task has a better execution performance and the scheduling completion degree is above 90%. The overall performance is minimally affected by how crowded the generated task set and the probability of high-criticality tasks entering HC mode.

It can be seen that the FDMC scheme proposed in this paper has obvious advantages in the performance of low-criticality tasks in the same mixed-critical system compared with the other three schemes in terms of completion degree and stability.

**Figure 8:**
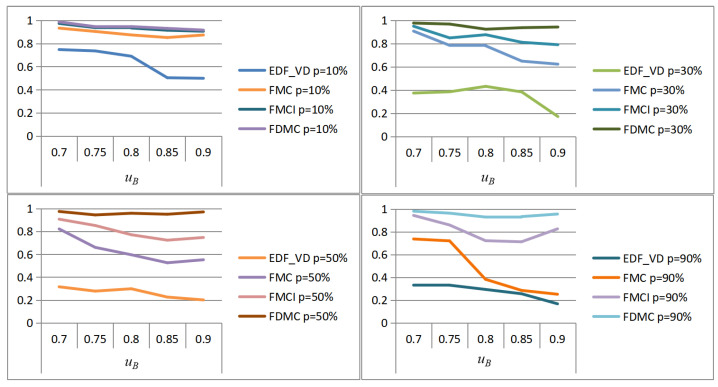
The PFJ comparison of different schemes under the same *p* value.

Figure 8 shows the horizontal comparison results of the PFJs of each scheme when the probability of the high-criticality task entering HC mode is the same (taking *p* = 10%, 30%, 50%, and 90% as an example). When p is 10%, the performance of FDMC and FMCI for low-criticality tasks is comparable, the performance of FMC is slightly worse, and the performance of EDF-VD has a certain lag gap compared with the other three schemes. When p increased, the differences between FMC, FMCI, and FDMC begin to appear gradually and the performance of low-criticality tasks under EDF-VD is the worst. When *p* is 90%, the performance gap of low-criticality tasks between FMC, FMCI, and FDMC is widened and the execution completion degree of low-criticality tasks under FMC is greatly reduced. We found that as the probability of high-criticality tasks entering HC mode is greater, the advantages of using FDMC for task scheduling in the performance of low-criticality tasks are more obvious. Low-criticality tasks under FMDC can also maintain a good degree of completion, when the task set is relatively congested in system scheduling.

Frequent mode switching will generate significant energy consumption, which may lead to problems such as system heating and performance degradation. Therefore, switching overhead is also a key consideration in task scheduling. Since the mode switching of high-criticality tasks in the EDF-VD scheme is not independent (when any high-criticality task overruns, all high-criticality tasks will perform mode switching), such switching costs must be the largest; here only the Independent mode switching of FMC, FMCI, and FDMC is evaluated. The switching cost mentioned here refers to the ratio of the number of mode switches performed by a high-criticality task to the number of tasks that are finally scheduled. That is, the fewer the number of mode switches (in the case of successfully scheduling the same number of tasks) or the larger the number of tasks that are finally successfully scheduled (in the case of the same number of mode switches), the lower the mode switching cost. This also means that the less unnecessary switching overhead, the greater the solution’s ability to avoid mode switching.

**Figure 9:**
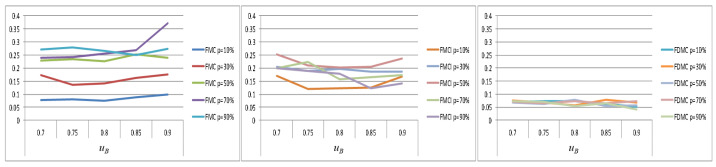
Evaluation of switching cost of each scheme under different *p* values.

Figure 9 shows the simulation results of the switching cost of the four schemes when a high-criticality task enters HC mode with different probabilities. Under FMC, the switching cost is greatly affected by the probability of high-criticality tasks entering HC mode and the switching costs under different probabilities are significantly different. The maximum mode switching cost exceeds 0.35, and the minimum is below 0.1. Compared with FMC, the switching cost under FMCI is significantly less affected by the probability of high-criticality tasks entering HC mode and the gap between switching costs under different probabilities is significantly narrowed. The mode switching cost is not more than 0.3 at the maximum, but is above 0.1 at the minimum. In FDMC, the mode switching cost is the least affected and the difference is not obvious under different probabilities, which are kept below 0.1.

Compared with other schemes, FDMC has obvious advantages in avoiding mode switching. The reason is that FDMC adopts dynamic allocation of the execution time overhead of high-criticality tasks in LC mode according to the actual operation of high-criticality tasks. That is, the condition for switching a high-criticality task to HC mode is no longer based on not being completed within its estimated execution time budget in LC mode, but by the maximum execution time budget allocated by dynamic calculation in its LC mode. Using FDMC for task scheduling can enable system resources to be called more flexibly and realistically, reduce unnecessary mode switching, and support the execution of more low-criticality tasks.

**Figure 10:**
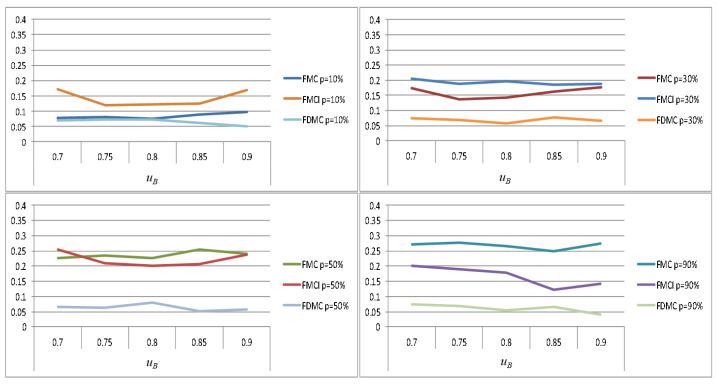
Horizontal comparison of switching cost results of each scheme under the same *p*.

Figure 10 shows the horizontal comparison of the switching cost of each scheme when the high-criticality task enters HC mode with the same probability (taking p = 10%, 30%, 50%, and 90% as examples). When p is 10%, the switching cost of FMCI is the largest, followed by FMC; the switching cost of FDMC is the smallest. As p increases, the switching costs of FMCI and FMC both increase to varying degrees, while the switching costs of FDMC does not change significantly. When p is 90%, the FDMC scheme still maintains a small mode switching overhead.

From the above comparison results, we can conclude that FMC is most affected by the probability of high-criticality tasks entering HC mode, followed by FMCI, while FDMC is the least affected. The FMC scheme performs well when the high-criticality task enters HC mode with low probability, but the switching cost increases significantly as the probability increases. Although the switching cost of the FMCI scheme does not change as much as the FMC, it still has a certain gap compared with the FDMC scheme. As the probability of a high-criticality task entering HC mode increases, the advantage of using the FDMC scheme for task scheduling in terms of switching cost performance becomes more obvious. Even if the task set is relatively congested in scheduling, the FMDC scheme can stably maintain a small switching cost.

## 7. Conclusions

In mixed-critical systems, traditional classical task scheduling methods often fail to achieve reasonable resource allocation and performance. In order to ensure the schedulability of high-criticality tasks, existing task scheduling schemes of mixed-criticality systems have problems such as waste of system resources and performance degradation of low-criticality tasks in pessimistic processing. This paper proposes a more practical method for self-adaptive adjustment of service levels for low-criticality tasks and a more realistic task scheduling scheme for dynamic mixed-criticality systems. The system dynamically allocates the execution overhead according to the actual operation of the high-criticality task in LC mode, avoiding unnecessary pessimistic processing in the existing task scheduling scheme, solving the problem of unnecessary extra execution budget resources generated by the static allocation method, and realizing the optimization of the task schedulability and system switching cost of a mixed-criticality system. Numerical results show that the service level of low-criticality tasks is improved while ensuring the schedulability of high-criticality tasks.

In terms of future work, we are interested in implementing the proposed method on a real-time operating system and evaluating its performance. In the process of dynamically allocating execution overhead, when the probability of high-criticality tasks entering HC mode is small and the total execution overhead of all high-criticality tasks in LC mode is less than the maximum execution budget threshold, there may still be room for further reclaiming idle system resources and improving task execution performance. In another research direction, it is possible to consider integrating FDMC and fault-tolerant technologies to develop better resource allocation strategies to ensure different fault types, since mixed-criticality systems have certain requirements for real-time and reliability.

## Figures and Tables

**Table 1:** Parameter information of task set.

$\tau_{i}$	$L_{i}$	$D_{i}$	$C_{\mathrm{il}}$	$C_{\mathrm{ih}}$	$D_{i}^{{}^{\prime }}$
$\tau_{1}$	LO	86	12	12	86
$\tau_{2}$	HI	51	6	12	29
$\tau_{3}$	HI	106	14	28	61
$\tau_{4}$	HI	30	3	6	17
$\tau_{5}$	LO	137	17	17	137
$\tau_{6}$	LO	145	20	20	145

## Data Availability

Not applicable.
